# Porcine Astrovirus Type 3 in Central Nervous System of Swine with Polioencephalomyelitis

**DOI:** 10.3201/eid2312.170703

**Published:** 2017-12

**Authors:** Bailey Arruda, Paulo Arruda, Melissa Hensch, Qi Chen, Ying Zheng, Chenghuai Yang, Igor Renan Honorato Gatto, Franco Matias Ferreyra, Phil Gauger, Kent Schwartz, Laura Bradner, Karen Harmon, Ben Hause, Ganwu Li

**Affiliations:** Iowa State University, Ames, Iowa, USA (B. Arruda, Q. Chen, Y. Zheng, C. Yang, F.M. Ferreyra, P. Gauger, K. Schwartz, L. Bradner, K. Harmon, G. Li);; Veterinary Resources Inc., Ames (P. Arruda);; The Maschhoffs, Carlyle, Illinois, USA (M. Hensch);; São Paulo State University (Unesp), Jaboticabal, Brazil (I.R.H. Gatto);; Cambridge Technologies, Worthington, Minnesota, USA (B. Hause)

**Keywords:** Mamastrovirus, swine, *Sus scrofa*, high-throughput nucleotide sequencing, neurologic disease, central nervous system, viruses, polioencephalomyelitis, pigs, astrovirus, meningitis/encephalitis, zoonoses

## Abstract

Using next-generation sequencing, we identified and genetically characterized a porcine astrovirus type 3 strain found in tissues from the central nervous system of 1 piglet and 3 sows with neurologic signs and nonsuppurative polioencephalomyelitis. Further studies are needed to understand the potential for cross-species transmission and clinical impact.

Astroviruses have been identified in a variety of mammals and birds; infection is often asymptomatic (*1*). Recently astroviruses have been implicated in cases of encephalomyelitis in humans, mink, cattle, and sheep (*2*‒*5*). We describe the use of unbiased next-generation sequencing to identify and genetically characterize a porcine astrovirus type 3 (PoAstV-3) in central nervous system (CNS) tissues of a 5-week-old piglet and 3 sows with neurologic signs and histopathologic lesions compatible with a neurotropic viral infection.

A multisite swine production farm submitted swine neurologic cases on 3 different occasions over a 9-month period to the Iowa State Veterinary Diagnostic Laboratory (Ames, Iowa, USA); 1 submission (2 live piglets) represented a population of 4–12-week-old pigs and 2 submissions (submission 2, two live sows; submission 3, head and tissue of sow) representing sows. In all cases, affected swine exhibited clinical signs that ranged from hind limb weakness to quadriplegia and occasionally convulsions (Video). The sow farm reported a case-fatality rate of 100%. The young pigs, which were farrowed from sows from the aforementioned sow farm, originated from 2 commercial grow-out facilities that reported a case-fatality rate of 75%. Histologic lesions in the CNS were consistent with a viral etiology. The following viruses were not detected in CNS samples by PCR: porcine reproductive and respiratory syndrome virus types 1 and 2, porcine circovirus 2, suid alphaherpesvirus 1, teschovirus A, sapelovirus A, or atypical porcine pestivirus. No pathogens were isolated by bacterial culture. Because of the persistence and severity of clinical signs, histologic lesions, and lack of detection of a viral etiology, two 5-week-old piglets and 4 sows with neurologic signs were submitted by a veterinarian for diagnostic testing by histopathology and next-generation sequencing. Histologic examination revealed severe, nonsuppurative polioencephalomyelitis in 3 of 4 sows and 1 of 2 piglets (Technical Appendix Figure).

**Video:**
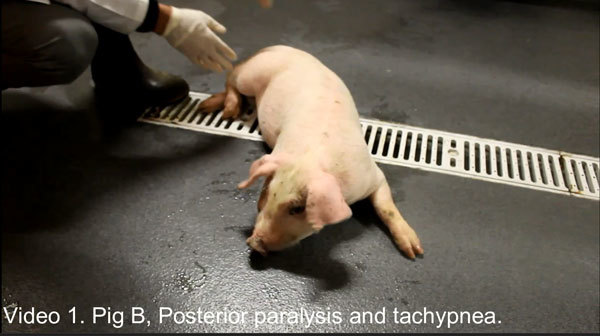
Posterior paralysis and tachypnea in pig infected with porcine astrovirus type 3.

We performed metagenomic sequencing for each animal using pooled RNA extracted from the cerebrum, cerebellum, brain stem, and spinal cord as previously described (*6*,*7*). We analyzed the sequences obtained using the MiSeq System (Illumina, San Diego, CA, USA) by using Kraken, an ultrafast and highly accurate program for assigning taxonomic labels by examining the k-mers within a read and querying a standard Kraken database with those k-mers (*8*). We assembled reads de novo using CLC Genomics Workbench (QIAGEN, Valencia, CA, USA) and identified the contigs by blastn (https://blast.ncbi.nlm.nih.gov/Blast.cgi). The largest contig, encompassing ≈2,000 reads, encoded a near-complete astrovirus genome of 6,461 nt and was designated PoAstV3/USA/IA/7023/2017 (GenBank accession no. KY940545). This sequence originated from a sow sample. A near-complete astrovirus genomic sequence was also obtained from the piglet (contig length 5,935 bp; E = 0) and had 100% nucleotide identity to PoAstV3/USA/IA/7023/2017. We also identified porcine endogenous retrovirus (contig lengths 1,865 bp and 1,317 bp; E = 0) in sow samples. When using a minimum contig length of 500 nt, we identified rocilivirus (contig length 832 bp; 32 reads; E = 0; GenBank accession no. KU058672.1) in piglet samples.

Phylogenetic comparisons of the capsid protein sequence, RNA-dependent RNA polymerase protein sequence, and whole-genome nucleotide sequence placed PoAstV3/USA/IA/7023/2017 in the same cluster as other strains of PoAstV-3 (Figure, panels A–C). The isolate we identified was most closely related to PoAstV3/USA/US-MO123 (GenBank accession no. NC_019494.1; 94.1% amino acid identity; Technical Appendix Table 1), which was detected in a swine fecal sample (*9*). On the basis of these phylogenetic analyses, PoAstV3/USA/IA/7023/2017 is more closely related to neurotropic astroviruses from humans, minks, cows, and sheep (*2*‒*5*) than to PoAstV-1, PoAstV-2, PoAstV-4, and PoAstV-5.

**Figure:**
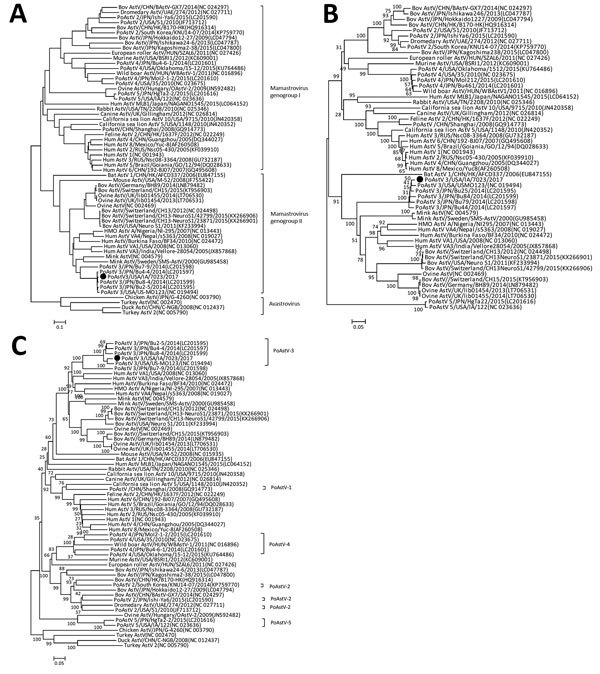
Phylogenetic trees of capsid protein (A), RNA-dependent RNA polymerase protein (B), and whole-genome nucleotide (C) sequences of a PoAstV type 3 strain (PoAstV3/USA/IA/7023/2017, filled circle) from central nervous system tissues of sows with neurologic signs and histopathologic lesions compatible with neurotropic viral infection compared with 66 reference viruses available in GenBank (accession numbers shown in parentheses), which came from multiple animal species (as indicated). We performed alignment using the Muscle model and constructed phylogenetic trees using the neighbor-joining method in MEGA6 (http://www.megasoftware.net). Virus types are labeled. AstV, astrovirus; PoAstV, porcine astrovirus. Scale bars indicate nucleotide substitutions per site.

We detected viral RNA by using a PoAstV-3 quantitative real-time PCR with previously fresh-frozen CNS tissues from animals with polioencephalomyelitis (Technical Appendix Table 2). We did not detect viral RNA in serum, feces, lung, liver, kidney, or spleen samples of animals with histologic lesions or any sample from animals without histologic lesions (*9*).

We describe the identification and genetic characterization of PoAstV-3 in CNS tissue from a piglet and sows with neurologic signs and histologic lesions compatible with a neurotropic virus similar to those described in neurotropic astrovirus cases in other species (*2*‒*5*). In humans, disease is primarily associated with immunocompromised patients. In cows, the virus is not commonly detected in feces, and the disease does not appear to be associated with immunocompromised animals (*4*). In this case, PoAstV-3 was not detected in feces of affected animals, and evidence of immunosuppression was lacking. The overall PCR prevalence of PoAstV-3 in feces of pigs in North America is reported to be low (1.2%) (*10*).

The PoAstV-3 we identified had 92.2% nucleotide sequence similarity to PoAstV-3 identified from a survey that evaluated feces samples from pigs (*9*).The significance of this finding is unclear. Investigations are needed to clarify the ecology and epidemiology of PoAstV-3 and the pathophysiology of neurotropic astroviruses. Studies have demonstrated the potential for recombination between porcine and human astroviruses, suggesting zoonotic potential (*9**,**10*).

Technical Appendix Description of tissue processing, amino acid differences between 2 porcine astrovirus isolates, porcine astrovirus type 3 quantitative real-time PCR results, and hematoxylin eosin stain of neuronal tissue from affected and nonaffected swine.
