# Supplementary material for: Porcine Astrovirus Type 3 in Central Nervous System of Swine with Polioencephalomyelitis
Source: Emerg Infect Dis. 2017 Dec;23(12):2097–100. doi: 10.3201/eid2312.170703 (PMC5708247; doi:10.3201/eid2312.170703)
Supplement: Technical Appendix — Description of tissue processing, amino acid differences between 2 porcine astrovirus isolates, porcine astrovirus type 3 quantitative real-time PCR results, and hematoxylin eosin stain of neuronal tissue from affected and nonaffected swine. [file 17-0703-Techapp-s1.pdf]

Article DOI: https://doi.org/10.3201/eid2312.170703

Porcine Astrovirus Type 3 in Central Nervous System of Swine with Polioencephalomyelitis

Technical Appendix

Additional Methods

Tissues were routinely processed at the Iowa State University Veterinary Diagnostic Laboratory for histologic examination. Central nervous system tissues (cerebrum; cerebellum; brainstem; and cervical, thoracic, and lumbar spinal cord) from each animal were independently reviewed by 2 veterinary diagnostic pathologists.

Technical Appendix Table 1. Amino acid differences between PoAstV3/USA/IA/7023/2017 and PoAstV3/USA/US-MO123/2011*

Genomic region, nucleotide position	Porcine astrovirus isolate, aa	
	USA/IA/7023/2017	USA/US-MO123
ORF1ab		
10	K	R
15	T	A
18	T	V
25	L	S
37	D	N
89	D	E
94	D	E
108	Y	W
112	K	N
122	L	S
129	K	R
132	Y	H
148	V	A
160	I	A
170	L	M
177	H	Y
216	D	G
217	G	D
230	F	L
295	F	L
300	F	L
320	I	T
413	R	K
473	T	P
523	A	V
586	R	K
610	S	N
657	H	R
694	L	F
713	T	A
748	A	T
827	K	R
856	H	Y
865	G	E

Genomic region, nucleotide position	Porcine astrovirus isolate, aa	
	USA/IA/7023/2017	USA/US-MO123
913	A	T
944	K	R
959	T	A
993	K	R
1024	E	V
1074	D	N
1075	A	D
1110	K	R
1196	C	R
1223	I	T
1334	R	G
ORF2		
17	I	T
23	I	T
24	S	F
25	L	W
44	F	S
57	E	A
68	P	L
107	S	N
114	R	K
120	N	S
197	D	G
251	S	N
333	K	Q
348	I	L
355	A	V
415	A	T
417	E	G
489	H	Y
496	A	V
497	G	N
499	T	N
501	S	T
502	P	H
504	S	P
513	L	K
519	N	D
528	S	P
531	G	P
556	S	A
576	D	N
585	N	A
601	N	D
614	N	E
633	S	N
635	N	D
644	D	N
658	T	S
663	H	S
684	A	T
707	L	P
710	T	A
711	A	V
725	T	N
726	F	Y
738	F	S
752	H	Q
763	N	Y

*ORF, open reading frame.

Technical Appendix Table 2. Porcine astrovirus type 3 quantitative real-time PCR results, by animal and sample type*

Animal ID	Histologic lesions†	Sample type, Cq				
		Cerebrum	Cerebellum	Cervical SC	Thorcic SC	Lumbar SC
Sow A	+	30.33	29.97	24.30	27.28	32.01
Sow B	–	U	U	U	U	U
Sow C	+	30.06	25.76	22.43	27.92	29.63
Sow D	+	U	U	34.92	35.87	38.61
Piglet A	–	U	U	U	U	U
Piglet B	+	30.77	ND	22.58	23.05	27.09

*Cq, quantification cycle value; ID, identification; ND, not done; SC, spinal cord; U, undetectable after 40 cycles.
†Presence (+) or absence (–) of polioencephalomyelitis.

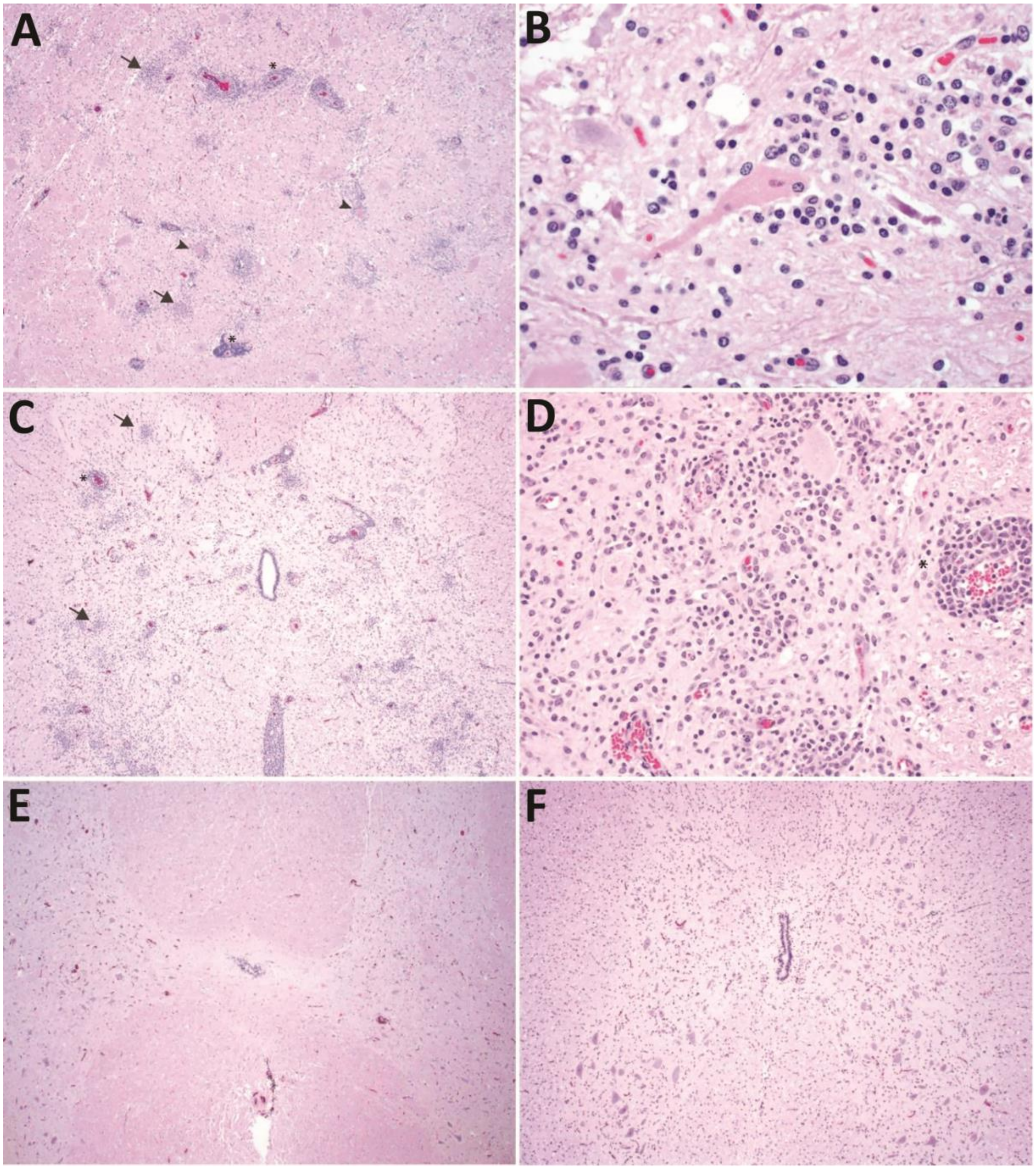

Technical Appendix Figure. Hematoxylin eosin stain of neuronal tissue of swine infected with porcine astrovirus type 3. A) Brainstem from sow C with rhombencephalitis. B) Neuronal necrosis in the brainstem of sow A. C) Nonsuppurative myelitis in piglet B. D) Focal area of gliosis, neuronal satelliosis, and neuronal necrosis in the spinal cord of piglet B. E) Histologically normal spinal cord of sow B. F) Histologically normal spinal cord of piglet A. Asterisk indicates expansion of the Virchow-Robin spaces by lymphocytes and plasma cells. Arrows show multifocal areas of gliosis. Arrow heads show neuronal necrosis.
